# Far-red light photoacclimation in a desert *Chroococcidiopsis* strain with a reduced FaRLiP gene cluster and expression of its chlorophyll *f* synthase in space-resistant isolates

**DOI:** 10.3389/fmicb.2024.1450575

**Published:** 2024-09-12

**Authors:** Giorgia di Stefano, Mariano Battistuzzi, Nicoletta La Rocca, Vera M. Selinger, Dennis J. Nürnberg, Daniela Billi

**Affiliations:** ^1^Department of Biology, University of Rome Tor Vergata, Rome, Italy; ^2^Ph.D. Program in Cellular and Molecular Biology, Department of Biology, University of Rome Tor Vergata, Rome, Italy; ^3^Department of Biology, University of Padua, Padua, Italy; ^4^National Council of Research of Italy, Institute for Photonics and Nanotechnologies (CNR-IFN), Padua, Italy; ^5^Giuseppe Colombo University Center for Studies and Activities, University of Padua, Padua, Italy; ^6^Institute of Experimental Physics, Freie Universität Berlin, Berlin, Germany; ^7^Dahlem Centre of Plant Sciences, Freie Universität Berlin, Berlin, Germany

**Keywords:** *Chroococcidiopsis*, FaRLiP, space exploration, Chl *f* synthase, genetic manipulation

## Abstract

**Introduction:**

Some cyanobacteria can use far-red light (FRL) to drive oxygenic photosynthesis, a phenomenon known as Far-Red Light Photoacclimation (FaRLiP). It can expand photosynthetically active radiation beyond the visible light (VL) range. Therefore, it holds promise for biotechnological applications and may prove useful for the future human exploration of outer space. Typically, FaRLiP relies on a cluster of ~20 genes, encoding paralogs of the standard photosynthetic machinery. One of them, a highly divergent D1 gene known as *chlF* (or *psbA4*), is the synthase responsible for the formation of the FRL-absorbing chlorophyll *f* (Chl *f*) that is essential for FaRLiP. The minimum gene set required for this phenotype is unclear. The desert cyanobacterium *Chroococcidiopsis* sp. CCMEE 010 is unusual in being capable of FaRLiP with a reduced gene cluster (15 genes), and it lacks most of the genes encoding FR-Photosystem I.

**Methods:**

Here we investigated whether the reduced gene cluster of *Chroococcidiopsis* sp. CCMEE 010 is transcriptionally regulated by FRL and characterized the spectral changes that occur during the FaRLiP response of *Chroococcidiopsis* sp. CCMEE 010. In addition, the heterologous expression of the Chl *f* synthase from CCMEE 010 was attempted in three closely related desert strains of *Chroococcidiopsis*.

**Results:**

All 15 genes of the FaRLiP cluster were preferentially expressed under FRL, accompanied by a progressive red-shift of the photosynthetic absorption spectrum. The Chl *f* synthase from CCMEE 010 was successfully expressed in two desert strains of *Chroococcidiopsis* and transformants could be selected in both VL and FRL.

**Discussion:**

In *Chroococcidiopsis* sp. CCME 010, all the far-red genes of the unusually reduced FaRLiP cluster, are transcriptionally regulated by FRL and two closely related desert strains heterologously expressing the chlF010 gene could grow in FRL. Since the transformation hosts had been reported to survive outer space conditions, such an achievement lays the foundation toward novel cyanobacteria-based technologies to support human space exploration.

## Introduction

Cyanobacteria are oxygenic photosynthetic prokaryotes. For a long time, they had been thought to be limited to using visible light (λ = 400–700 nm, VL). However, a few strains, diverse in their morphologies and phylogenetic histories, have been discovered that are capable of expanding their photosynthetic capacity into far-red light (λ = 700–800 nm, FRL), by using a complex process known as far-red light photoacclimation (FaRLiP; Gan et al., 2014). Many such strains have been identified in shaded environments, which are attenuated in VL and enriched in FRL (e.g., Antonaru et al., 2020; Averina et al., 2018; Gan and Bryant, 2015; Gan et al., 2015; Sanfilippo et al., 2019; Zhang et al., 2019). During the acclimation process, FaRLiP cyanobacteria remodel their photosynthetic machinery and synthesize far-red absorbing chlorophylls (Chls), such as Chl *f* and Chl *d* (Gan et al., 2014). These changes depend on a cluster of FaRLiP genes that contains master control elements (RfpA/B/C), plus paralogs of the Photosystem I (PSI; PsaA2/B2/F2/I2/J2/L2), Photosystem II (PSII; PsbA3/A4/B2/C2/D3), and phycobilisomes (ApcB2/D2/D3/D5/E2; Gan and Bryant, 2015). Other FaRLiP-specific paralogs, such as PsbH2, may optionally be present (Gan and Bryant, 2015). Perhaps the most important gene in the cluster is *psbA4*, now known as *chlF*, a divergent D1 paralog that is not involved in water oxidation but rather in Chl *f* synthesis (Ho et al., 2016; Trinugroho et al., 2020).

Shading in the visible spectrum, accompanied by FRL enrichment, is a common constraint for desert cyanobacteria, as these organisms may colonize rocks to avoid UV radiation and desiccation (Pointing and Belnap, 2012). Most lithic substrates have FRL-shifted transmission spectra (Meslier et al., 2018; Smith et al., 2014). *Chroococcidiopsis* or related genera have been commonly reported from these environments (Friedmann, 1980; Bahl et al., 2011), and this genus has also been associated with FaRLiP (Antonaru et al., 2023; Murray et al., 2022). However, FaRLiP seems to be rare in deserts (Antonaru et al., 2023; Gwizdala et al., 2021). Later studies have highlighted that *Chroococcidiopsis* as previously understood is heavily polyphyletic and showed that FaRLiP is rare in extremophile genera within the same order as the current definition of the genus (Antonaru et al., 2023; Jung et al., 2021). The strains investigated in this study belong to one such extremophile genus, still untypified, that is specialized for life in deserts (Antonaru et al., 2023; Fagliarone et al., 2017). Within it, most strains do not have a FaRLiP cluster. An exception is *Chroococcidiopsis* sp. CCMEE 010 (hereafter referred to as CCMEE 010), isolated from subsurface growth within a rock from the Negev Desert. This strain can grow under FRL, but has a uniquely reduced FaRLiP cluster, devoid of most FRL-PSI genes (four paralogs out of six; Billi et al., 2022). It also lacks the optional *psbH2* gene found in strains from related genera such as *Chroococcidiopsis thermalis* PCC 7203 (Gan and Bryant, 2015).

Expanding photosynthetically active radiation into FRL by means of engineering biology approaches, has been considered as a strategy for improving photosynthetic yield in cyanobacteria and plants (Liu et al., 2021; Elias et al., 2021). However, it is debatable to what extent it could be applied at a large scale given the trade-offs inherent in this adaptation (Viola et al., 2022). Relying on FRL-shifted spectra through genetic manipulation of phototrophs may however prove useful for confined environments such as the International Space Station, or future outposts on the Moon or Mars (Jung et al., 2023; Liistro et al., 2024). The genetic modification of cyanobacteria to improve carbon uptake and oxygen output is considered an essential step toward sustainable and self-sufficient systems for human space exploration (Koehle et al., 2023). For example, an enhanced photosynthetic activity was achieved through metabolic engineering in two model cyanobacterial species, *Synechocystis* sp. PCC6803 and *Synechococcus elongatus* PCC7942 (Kamennaya et al., 2015; Santos-Merino et al., 2021). In parallel, the heterologous expression of the *chl*F gene in these cyanobacteria was performed, as a first step, necessary but not sufficient, for transferring FaRLiP (Ho et al., 2016; Shen et al., 2019; Trinugroho et al., 2020; Tros et al., 2020). However, these model strains are from non-extreme environments (Santos-Merino et al., 2019). To manage the constraints imposed by altered gravity and space radiation, an ideal component of life support systems for outer space would be cyanobacteria with increased photosynthetic efficiency coupled with resistance to extreme conditions (Koehle et al., 2023).

Previously, the FaRLiP process of CCMEE 010 was studied by assessing its capability to grow in FRL (Billi et al., 2022) and produce Chls *f* and *d* (Antonaru et al., 2023). However, the way it acclimates to FRL using a reduced cluster is not fully understood. Its FaRLiP gene cluster (15 genes as opposed to ~20) may prove of biotechnological interest for the development of new bioprocesses for space applications.

However, transferring FaRLiP between cyanobacteria has been a challenge due to the difficulties of transplanting a cluster of about 30 kbp (Luan et al., 2020). Therefore, a cyanobacterium with a reduced but functional FaRLiP cluster may help to overcome this limitation. A first step in this direction would be to transfer the *chlF* gene of CCMEE 010 to related and space-resistant *Chroococcidiopsis* strains CCMEE 029, CCMEE 057, and CCMEE 064. These desert strains are not capable of FaRLiP (Billi et al., 2022; Antonaru et al., 2023), but they are able to resist desiccation and ionizing radiation (Fagliarone et al., 2017; Verseux et al., 2017) and have also survived space conditions, as demonstrated by space experiments using a platform installed outside the International Space Station (Billi et al., 2019a,b). In addition, strains CCMEE 029 and CCMEE 057 are suitable for genetic manipulation (Billi et al., 2001).

The present work has two objectives: (i) to study the expression of the reduced gene cluster and spectral characteristics during FRL acclimation of CCMEE 010; and (ii) to confer the ability to grow in FRL to closely-related and space-relevant strains. Thus, the transcription levels of FaRLiP genes were investigated in CCMEE 010 cells exposed for 48 h to FRL and changes in the spectral features of the photosynthetic apparatus were determined during a 21-day growth period in FRL. A synthetic plasmid carrying the *chl*F gene of CCMEE 010 was designed and used for genetic manipulation of strains CCMEE 029, CCMEE 057, and CCMEE 064. The transformants were selected in FRL and VL conditions (combined with antibiotic pressure) and investigated for both the expression of the *chl*F^010^ gene and alterations of the spectral features of the photosynthetic apparatus.

## Materials and methods

### Microorganisms and growth conditions

Four *Chroococcidiopsis* strains were obtained from the Culture Collection of Microorganisms from Extreme Environments (CCMEE) established by E. Imre Friedmann and Roseli Ocampo-Friedmann and currently maintained at the Department of Biology, University of Tor Vergata. Strains CCMEE 010 and CCMEE 029 were isolated from rock samples collected in the Negev Desert and strains CCMEE 057 and CCMEE 064 from the Sinai Desert. Cyanobacterial strains were grown without shaking in liquid BG-11 medium at 25°C under a constant photon flux density of 40 μmols of photons m^2^ s^−1^, provided with a fluorescent cool-white lamp (OSRAM L 8W/640, Osram, Germany), or with a 750 nm LEDs (Epitex; L750-01AU). *Escherichia coli* DH5α was grown in Luria-Bertani (LB) medium at 37°C under orbital shaking. Growth media were supplemented with ampicillin (50 μg ml^−1^) as required.

### Confocal laser scanning microscopy

Cells of *Chroococcidiopsis* sp. CCMEE 029, CCMEE 057, and CCMEE 064 were immobilized with BG-11 medium containing 1.5% (w/v) agarose on the top of microscope slides and examined with a Confocal Laser Scanning Microscopy System (CLSM; Olympus Fluoview 1000). CLSM lambda scans were obtained by using a 488 nm excitation laser and detecting the emission from 500 to 799 nm. Fluorescence emissions curves were plotted based on the mean fluorescence intensity (MFI) using GraphPad Prism version 8.4.3 (GraphPad Software, San Diego, CA, USA).

### Transformation

The synthetic plasmid pTac-ChlF-pDU1 carrying the Chl *f* synthase gene of CCMEE 010 (GenBank accession OM156469; hereafter chlF^010^) was generated by GenScript Biotech (Rijswijk, Netherlands). The plasmid was maintained in *E. coli* DH5α and extracted from overnight cultures grown at 37°C in LB medium supplemented with 50 μg ml^−1^ ampicillin, by using the Zymo Pure^TM^ Plasmid Miniprep Kit (Zymo Research Europe). For electroporation, aliquots of CCMEE 029, CCMEE 057, and CCMEE 064 were washed twice with cold 1 mM HEPES buffer, pH 7.4, and resuspended in 50 μl of the same buffer (containing about 10^8^ cells), then 2.5 ng μl^−1^ of purified plasmid were added to the cyanobacterial suspension and electroporated as described previously (Billi et al., 2001). After electroporation 950 μl of BG-11 medium was added and the cells were incubated under the aforementioned conditions for 24 h. Then 5-μl aliquots were spotted on Whatman^®^ nitrocellulose membrane filters on BG-11 medium solidified with 1.5 % (w/v) agar and containing 50 μg ml^−1^ ampicillin. Transformant selection was performed under VL (4 μmol photons m^−2^ s^−1^) or FRL (40 μmol photons m^−2^ s^−1^). Due to the slow generation time of 4–5 days the transformants were not restreaked onto fresh medium, but after 15 days ampicillin (50 μg ml^−1^) was added to the top of the solidified BG-11 medium until cells were collected and analyzed as reported below.

### Isolation of total RNA and gene transcription analysis

Total RNA was extracted using the RNeasy^®^ Micro Kit (Qiagen, Inc.) from CCMEE010 after 48 h growth under FRL and from control cells grown under VL. The extracted RNA was used for first-strand cDNA synthesis by using the SensiFAST^TM^ cDNA Synthesis Kit (Bioline Meridian Life Science Memphis, TN, USA), which then was used as a template for RT-qPCR. Reactions were performed in 12 μl reaction volumes including 1 μg of cDNA as template, 6 μl of iTaqTM universal SYBR^®^ Green supermix (BioRad Laboratories, Hercules, CA, USA) and 0.5 nM of forward and reverse primers designed to target each one of the 15-FaRLiP genes (Supplementary Table 1). The 16S rRNA gene was used for normalization and amplified by using primers 16SF (5′- TACTACAATGCTACGGACAA-3′) and 16SR (5′- CCTGCAATCTGAACTGAG-3′) that target the 16S rRNA gene of CCMEE 029, CCMEE 057 and CCMEE 064 (GenBank accession numbers: AF279107, AF279108 and AY301001). PCR cycling conditions were performed using a StepOnePlus^TM^. Real-Time PCR System (Thermo Fisher Scientific, Waltham, MA, USA) as follows: one step at 95°C for 10 min, followed by 40 cycles of 95°C for 15 s and 60°C for 1 min, and a ramp from 60 to 95°C for the melting curve stage. Relative mRNA levels were calculated by the comparative cycle threshold (Ct) method and the 16S rRNA gene was used for normalization. Fold-change values were calculated by setting the values obtained from VL-grown cells as 1 and considering the values from FRL-grown cells as upregulated (>1) or downregulated (<1). For each gene target more than 3 RT-qPCR reactions were conducted, each one in triplicate.

Total RNA was extracted from 1-month old transformants of CCMEE 029 CCMEE 057 and CCMEE 064 obtained as reported above and from wild-type strains. RT-qPCR were performed as described above, by using the forward and reverse primers d chlF^010^ gene (Supplementary Table 1). Fold-change values were calculated by setting the values obtained from wild-type strains as 1 and considering the values from transformants as upregulated (>1) or downregulated (<1).

## Results

### Overexpression of FaRLiP genes during the FRL response in CCMEE 010

**Figure 1:**
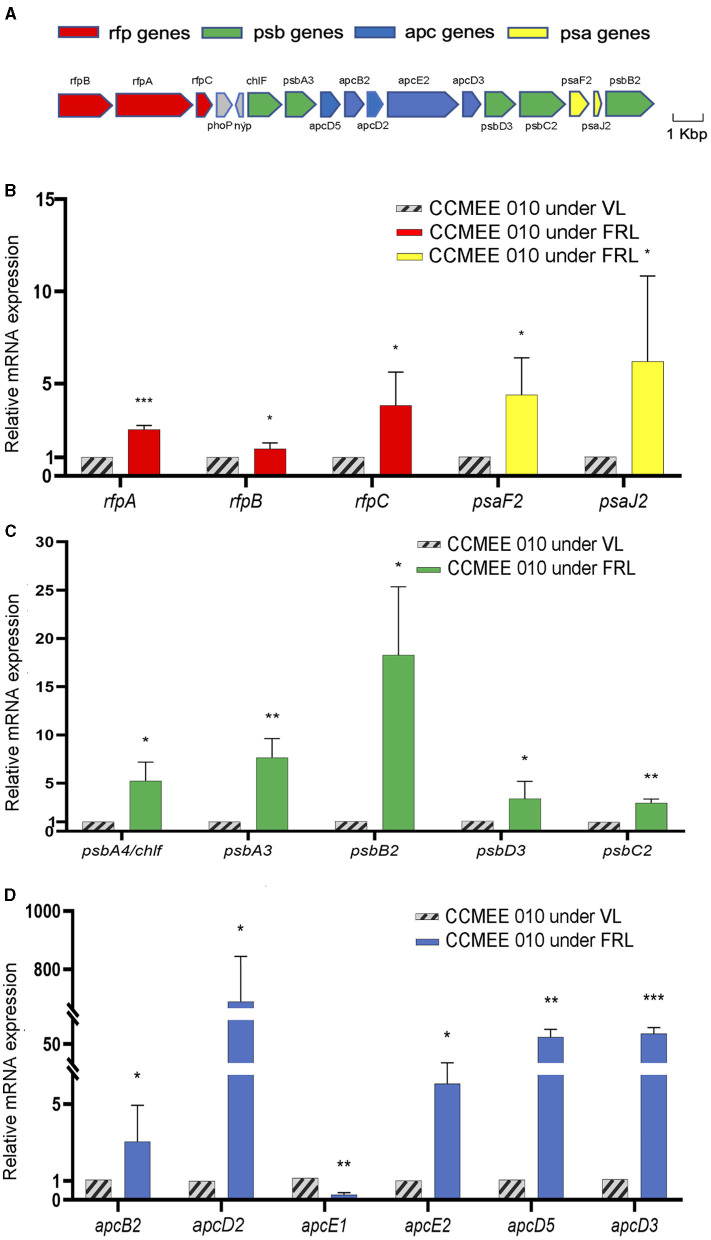
Gene expression of the FaRLiP cluster in FRL-acclimated CCMEE 010. Structure of far-red gene cluster **(A)**, color-coding: *psa* genes of FR-PSI (yellow); *psb* genes of FR-PSII (green); *apc* genes of FR-phycobilisomes (blue); *rfp* genes (red); Relative expression after 48 h-exposure to FRL as revealed by RT-qPCR of the *rfpA, rfpB*, and *rfpC* genes and FRL-paralogs of the PSI **(B)**, PSII **(C)**, and phycobilisomes **(D)**; The *apcE1* gene was used as a control gene highly expressed only in VL condition. Data represents mean +/- SD. Statistical significance for each gene in FRL- vs. VL-cells, was assessed by *t*-test and indicated by ***P < 0.001; **P < 0.01; and *P < 0.05.

The transcriptional changes of the 15-FaRLiP genes of CCMEE 010 were investigated after 48 h exposure to FRL (Figure 1). The respective FaRLiP cluster is shown in Figure 1A. It contains three components of the phytochrome signaling cascade (*rfpA/B/C)*, two FRL-PSI paralogs (*psaF2/J2)*, five FRL-PSII paralogs (*psbA3/A4/B2/C2/D3*) and five FRL-phycobilisomes paralogs (*apcB2/D2/D3/D5/E2*) as previously reported (Billi et al., 2022).

Relative transcript levels increased for all of the genes in the reduced FaRLiP cluster when kept for 48 h under FRL. The transcript levels of the *rfpA, rfpB*, and *rfpC* genes were increased by 2.5, 1.5-, and 3.8-fold compared to VL-control (Figure 1B). The transcript abundances of the two FRL-PSI paralogues, *psaF2* and *psaJ2* genes, were increased by 4.4- and 6.2-fold with respect to VL-control (Figure 1B). Increased transcript levels of FRL-PSII paralogues were detected in FRL cells. The *psbA4/chlF* gene showed a 5.5-fold increase, the *psbA3* and *psbD3* genes encoding the D1 and D2 FR-paralogues, were 7.7- and 3.4-fold increased, respectively, while the expression levels of the *psbB2* and *psbC2* genes encoding CP47 and CP43, were 18.3-, and 3-fold increased, respectively (Figure 1C). The expression of the *apcE2* gene encoding a phycobilisome-membrane linker was 6-fold increased (Figure 1D). The transcripts of *apcB2, apcD2, apcD3*, and *apcD5* genes encoding red-shifted allophycocyanin subunits, were about 3-, 680, 72- and 64-fold more abundant in FRL cells than in VL-control (Figure 1D). As expected FRL-cells showed a reduced expression level of the *apcE1* gene, typically expressed only in VL-cells (Zhao et al., 2015; Figure 1D).

### Spectroscopic features of CCMEE 010 during FRL-acclimation

**Figure 2:**
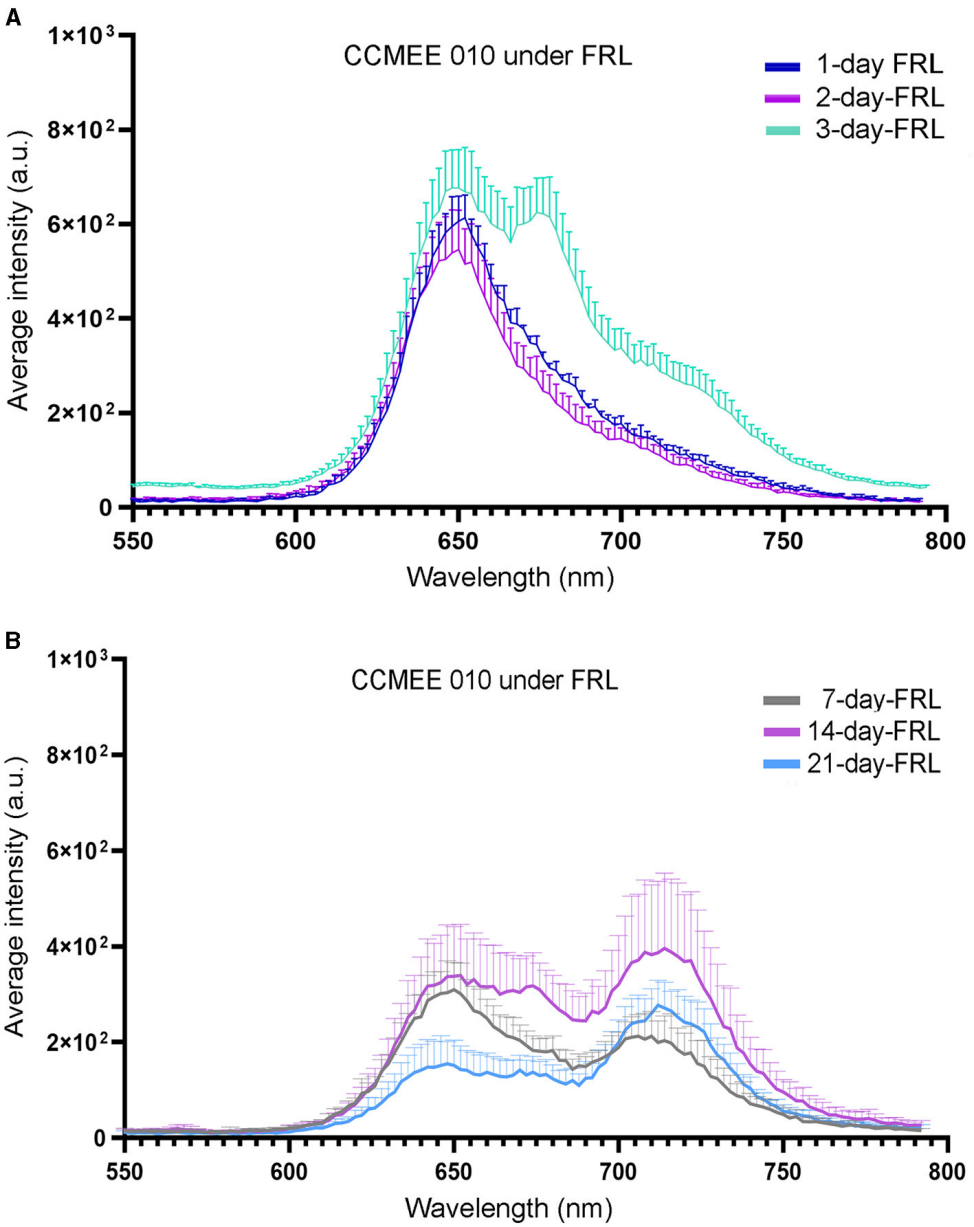
CLSM-lambda-scan analysis of single cells of FRL-acclimated CCMEE 010. Spectra of FRL-exposed cells for 1, 2, and 3 days **(A)** and for 7, 14, and 21 days **(B)**. Data points represent normalized mean fluorescence intensity at 653 nm +/– standard error for *n* = 20 cells as a function of emission wavelength.

The spectral characteristics of single cells of CCMEE 010 exposed to FRL were investigated by CLSM-lambda-scan analysis (Figure 2). After 1 and 2 days in FRL, cells still showed a fluorescence emission peak in the 640–667 nm range (Figure 2A), typical of VL-grown cells, due to phycobiliproteins and Chl *a*-containing photosystems. After 3 days in FRL, an additional peak at around 672–679 nm appeared (Figure 2B), that after 7 days in FRL was replaced by a FR-shifted (715–727 nm) peak. The latter is due to Chl *f* and FRL-phycobiliproteins, as previously reported for CCMEE 010 grown for 14 days in FRL (Billi et al., 2022).

### Space-resistant strains of *Chroococcidiopsis* transformed with the *chl*F^010^ gene

**Figure 3:**
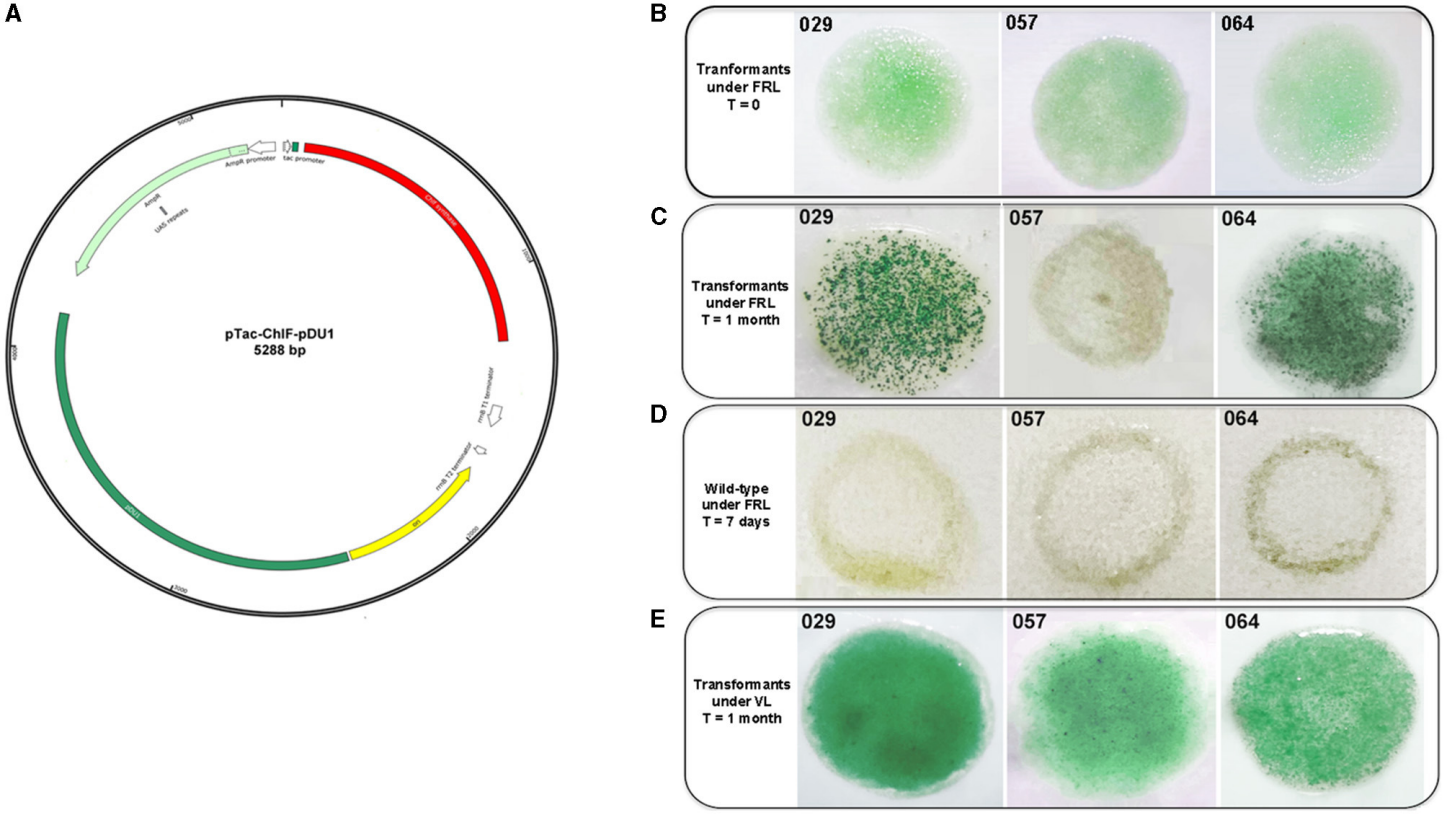
Transformation of CCMEE 029, CCMEE 057, and CCMEE 064. Map of pTac-ChlF-pDU1 plasmid **(A)**; Transformants selection under FRL and antibiotic pressure, *t* = 0 days after electroporation **(B)**; Transformants selection under FRL and antibiotic pressure after 1 month **(C)**; Wild-type strains under FRL **(D)**; Transformants selection under VL and antibiotic pressure **(E)**.

The genetic manipulation of the strains CCMEE 029, CCMEE 057 and CCMEE 064 was performed via electroporation by using the synthetic plasmid pTac-ChlF-pDU1. This plasmid carried the *chlF*^010^ gene downstream of the P_*tac*_ promoter, an *E. coli* origin of replication and the pDU1 sequence, known to maintain plasmid replication in CCMEE 029 and CCMEE 057 (Billi, 2012; Figure 3A).

Transformants were selected in FRL condition by spotting the electroporated cells on the top of solidified BG-11 medium containing ampicillin (Figure 3B). After 1 month, intense green spots of transformants occurred for both CCMEE 029 and CCMEE 064, whereas the spots for CCMEE 057 bleached within 1 week (Figure 3C). Wild-type strains bleached within 1 week after being spotted onto BG-11 without ampicillin and incubation in FRL condition (Figures 3C, D).

Transformants were also selected in VL. After 1 month, intense green spots were obtained for each strain (Figure 3E), while untransformed wild-type strains bleached within 1 week in the presence of ampicillin (not shown).

### Expression of the *chl*F^010^ gene in *Chroococcidiopsis* transformants

**Figure 4:**
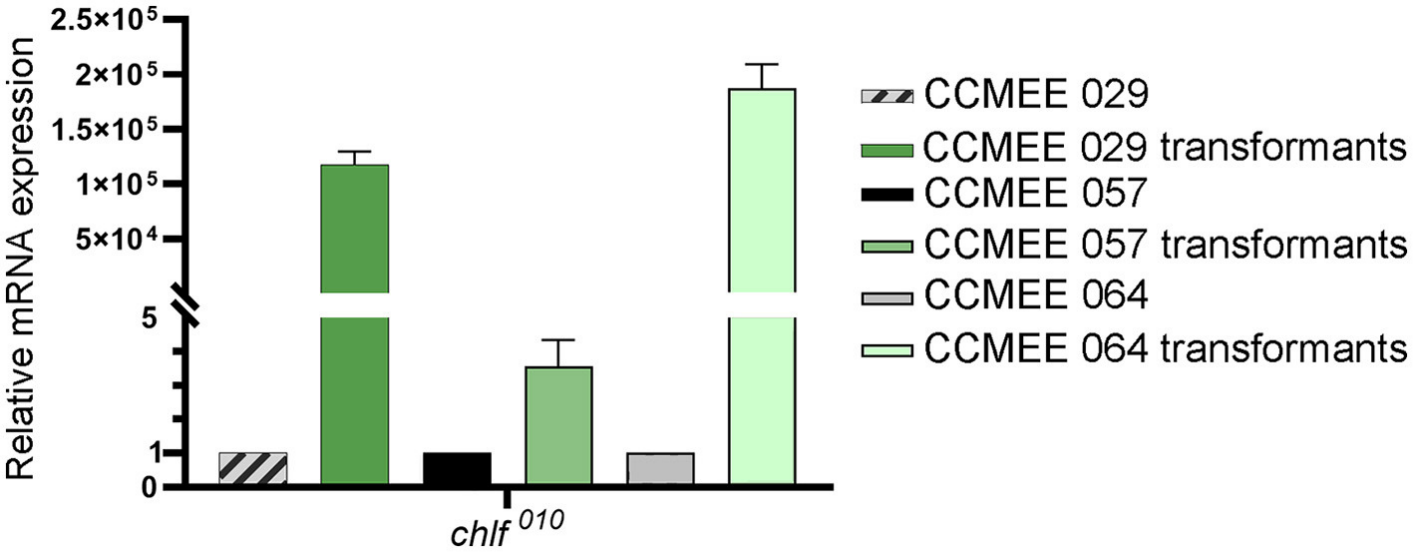
Heterologous expression of *chl*F^010^ gene in *Chroococcidiopsis* transformants under VL-condition. Transcription levels in transformants of strain CCMEE 029, CCMEE 057, and CCMEE 064.

The expression level of the *chl*F^010^ gene was determined in CCMEE 029, CCMEE 057 and CCMEE 064 transformants that were obtained after 1-month of incubation under VL and antibiotic pressure (Figure 3E). The pTac-ChlF-pDU1 plasmid contained the P_*tac*_ promoter upstream of the *chl*F^010^ gene, but lacked the *lacI* repressor common to many IPTG-inducible expression vectors, which resulted in constitutive expression as previously demonstrated (Billi, 2012). Transformants of CCMEE 029 and CCMEE 064 showed relatively high expression levels of the *chl*F^010^ gene (Figure 4), whereas lower transcript levels of the *chl*F^010^ gene occurred in CCMEE 057 transformants (Figure 4).

### Spectral features of *Chroococcidiopsis* transformed with *chlF*^010^

**Figure 5:**
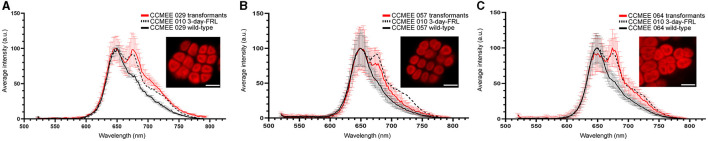
CLSM image and lambda scans of *Chroococcidiopsis* transformed with the *chl*F^010^ gene under VL-condition. Spectral fluorescence emissions of 1-month-old transformants CCMEE 029 **(A)**, CCMEE 057 **(B)**, and CCMEE 064 **(C)**, are shown as red lines; emission spectra of wild-type strains (black lines) and CCMEE 010 after 3 days in FRL (dotted lines). Data are mean fluorescence intensity ± standard deviation for *n* > 15 cells. CLSM images (optical sections) corresponding to the maximum emission peaks of the photosynthetic pigments of transformants excited at 488 nm. Scale bar = 5 μm.

One month after the electroporation and incubation under VL with antibiotic selection (Figure 3E), transformants of CCMEE 029, CCMEE 057 and CCMEE 064, were investigated for changes in their spectral features (Figure 5). The CLSM lambda scans at the single-cell level showed that transformants had a photosynthetic pigment emission spectrum with a peak at about 672–679 nm, that was absent in wild-type strains and resembling the ones detected in CCMEE 010 after 3 days in FRL (Figure 5). In particular, CCMEE 029 and CCMEE 064 transformants had a comparable intensity of the 672–679 nm peak (Figures 5A, C), which was slightly reduced in CCMEE 057 transformants (Figure 5B).

## Discussion

In this study, a first insight was gained into FRL-acclimation in the desert cyanobacterium *Chroococcidiopsis* sp. CCMEE 010, which to date is unique among FaRLiP cyanobacteria for having a reduced FaRLiP gene cluster that lacks four out of six PSI paralogs (Billi et al., 2022). Further information was obtained on the possibility of expressing the *chl*F^010^ gene to extend the photosynthetically active radiation of space-relevant strains closely related to CCMMEE 010.

The role played by the reduced FaRLiP gene cluster in driving the FRL-acclimation process of CCMEE 010 was revealed by the expression levels of the 15-FaRLiP genes, which increased from 1.5- up to 680-fold after 48h-exposure to FRL. This was overall in agreement with the expression levels of the 21-gene cluster of *Leptolyngbya* sp. strain JSC-1 exposed to FRL for 24 h (3- to 278-fold; Gan et al., 2014). The *rfpA, rfpB*, and *rfpC* genes encoding master control elements had the smallest increase in expression level (1.5–3.8 times). This is likely due to the need for these regulators to be expressed under VL as well in order to detect FRL and regulate expression of the FaRLiP cluster. While the largest changes (3–680 fold) occurred among the five genes encoding FRL-phycobilisomes (*apcB2, apcD2, apcD3, apcD5*, and *apcE2*; Billi et al., 2022), high levels of transcripts were found for the four genes encoding FRL-PSII subunits, namely the *psbA3, psbB2, psbC2*, and *psbD3* genes (Billi et al., 2022). The *chl*F gene was highly expressed, thus confirming its central role in the FaRLiP response (Gan et al., 2014; Ho et al., 2016), and in agreement with the lack in the encoded amino acid sequence, of the residues necessary for water oxidation (Billi et al., 2022).

Despite lacking most FRL-PSI paralogs (*psaA/B/L/I*), the transcription levels of the two remaining FRL-PSI genes, *psaF2* and *psaJ2*, were also increased. It has previously been hypothesized that PsaF2 and PsaJ2 play a role in the incorporation of Chl *f* molecules in PSI (Gisriel et al., 2020), and our data indirectly supports this. Due to the lack of the FRL-PSI reaction core subunits (*psaA/B*) in this organism, it is likely that the remaining FRL-PSI subunits are incorporated into VL photosystems, at least during the short acclimation period studied. No expression was observed for VL-genes *psaA* and *psaB* after 48 h-exposure to FRL (not shown), but this can be explained by the longer half-life times of PSI proteins (in the order of a couple of days) compared to the few-hour half-life of PSII proteins (Yao et al., 2012). Presumably, the known capacity of CCMEE 010 to grow photoautotrophically under FRL (Billi et al., 2022) is supported by a VL/FRL-PSI hybrid with Chl *a* and Chl *f*. The presence of VL components in CCMEE 010′s PSI could reinforce an adaptation mechanism as reported for FRL-acclimated cyanobacteria, which retain VL-harvesting complexes and gain the flexibility needed to absorb the VL as soon as it is available (Ho et al., 2020).

Compared to transcriptional changes, spectral changes occurred more slowly, with no obvious changes during the first 2 days. A new peak appeared at about 672–679 nm after 3 days, which after 7 days in FRL, was replaced by a FR-shifted peak at 715–727 nm, in agreement with the presence of Chl *f* and far-red allophycocyanin, as previously reported for CCMEE 010 after 14 days in FRL (Billi et al., 2022). This suggested that the remodeling of the photosystem apparatus in a cyanobacterium with a reduced FaRLiP cluster is as slow as in one with a complete FaRLiP cluster. For example, in *Chlorogloeopsis fritschii* PCC6912, a very small *in vivo* absorption of light beyond 700 nm was detected after 3 days in FRL, which became evident after 21 days (Battistuzzi et al., 2023).

The extension of photosynthetically active radiation of three space-relevant desert strains of *Chroococcidiopsis*, namely, CCMEE 029, CCMEE 057 and CCMEE 064, which are FaRLiP-incapable (Antonaru et al., 2023; Billi et al., 2022), was attempted via heterologous expression of the *chl*F^010^ gene. The fact that transformants of the three strains could be selected under VL (and antibiotic pressure) suggested that the cyanobacterial pDU1 replicon allowed the replication of the pTac-ChlF-pDU1 plasmid in each strain. On the contrary, only CCMEE 029 and CCMEE 064 transformants were obtained under FRL (and antibiotic pressure). Moreover, the fact that after 1-month incubation the cell spots showed a higher cell density, suggested the capability of growing in FRL. The failure to obtain CMEE 057 transformants in FRL might be due to a limited amount of Chl *f* synthase, as suggested by the low transcription level of the *chl*F^010^ gene compared to CCMEE 029 and CCMEE 064 transformants. The *chl*F^010^ gene expression was driven by the P_*tac*_ promoter, a derivative of the P*lac* promoter from the *lac* system of *E. coli*, based on IPTG induction and LacI-suppression (Gilbert and Müller-Hill, 1966). However, in the present study, P_*tac*_ was used as a constitutive promoter in the absence of *lacI* because IPTG-based induction systems have been reported to be leaky (a high baseline) or with a low-fold induction (Till et al., 2020; Gordon and Pfleger, 2018) in several cyanobacterial hosts. In addition, no differences were observed in the fluorescence signal of the green fluorescent protein driven by the P_*tac*_ promoter with or without IPTG induction in CCMEE 029 transformants (Billi, 2012). Therefore, the reduced transcription levels of the *chl*F^010^ gene in CCMEE 057 transformants compared to CCMEE 029 and CCMEE 057 transformants, could be due to a variation in the strength of P*lac* between strains, as reported for various cyanobacteria (Berla et al., 2013).

In this study, in order to detect Chl *f*, transformants were incubated at low VL-irradiance (4 μmol photons m^−2^ s^−1^) to minimize the turnover of light-activated Chl *f* synthase and to prevent photo-oxidation of Chl *f* (Ho et al., 2016; Shen et al., 2019). The transformants had a photosynthetic pigment emission spectrum with a peak at about 672–679 nm, which do not occur in wild-type strains but resemble that detected in CCMEE 010 after 3 days in FRL. However, unlike CCMEE 010 where after 7 days in FRL, the 672–679 nm was replaced by a FR-shifted peak at 715–727 nm, none of the transformants showed a far-red shifted peak. The fact that CCMEE 057 transformants showed a reduced intensity of the 672–679 nm peak, is in agreement with the reduced expression of the *chl*F^010^ gene and the failure to select CCMEE 057 transformants in FRL. The lack of red-shifted fluorescence in *Chroococcidiopsis* transformants may be due to uncoordinated insertion of Chl *f* in these photosystems but also to the absence of FRL-phycobilisomes components. In a *Synechococcus* sp. PCC 7002 mutant heterologously expressing a *chlF* gene, Chl *f* was functionally linked to the reaction center of the PSI complex, although the Chls *f* bound to PSI were not as red-shifted as in FaRLiP-cyanobacteria (Tros et al., 2020).

In this study, the presence of Chl *f* in *Chroococcidiopsis* transformants could not be supported by HPLC analysis (not shown), likely due to undetectable Chl *f* amounts in the 1-month-old transformant spots used for the analysis. This is in agreement with the fact that generally, FaRLiP cyanobacteria replace only 10% of Chl *a* with Chl *f* (Gan et al., 2014) and lower Chl *f* amounts occur in cyanobacteria heterologously expressing a *chlF* gene. The highest Chl *f* amount (3–4% of the total Chl content), corresponding to about 50% of the Chl *f* content in FarLiP strains, was reported for *Synechococcus* sp. PCC 7002 transformants (Shen et al., 2019). Lower Chl *f* levels (about 0.24% of Chl *a* content) also occurred in transformants of *Synechocystis* sp. PCC 6803 (Trinugroho et al., 2020). Furthermore, while in *Synechococcus* sp. PCC 7002 the *chlF* gene expression was driven by the strong P_*cpcBA*_ promoter derived from *Synechocystis* sp. PCC 6808, in the present work, a P_*tac*_ promoter has been used due to its previously reported activity in CCMEE 029 (Billi, 2012).

Although it is necessary to further investigate the photosynthetic efficiency of *Chroococcidiopsis* transformants, the possibility of extending the spectrum of light absorption beyond the red limit of two space-resistant strains has laid the foundations for new cyanobacteria-based technologies to support human space exploration. In this context, there is a growing interest in using lithotrophic cyanobacteria for the utilization of local materials on the Moon or Mars, the so-called *in-situ* resource utilization. Being the yielded biomass employed for several applications, like footstock for bacteria or biostimulant for plants (Verseux et al., 2016). However, the mixing with regolith might give rise to shaded environments enriched in FRL. Genetically engineered CCMEE 029′s cells with an extended photosynthetically active radiation are a suitable candidate for space bioprocesses based on *in-situ* resource utilization, since this strain can grow using minerals present in Moon and Mars regolith simulants (Fernandez et al., 2023). Space-tolerant strains with an extended photosynthetically active radiation could also open up other innovative avenues to support human exploration of deep space. An enhanced photosynthetic efficiency could be beneficial to long-term crewed missions, by enhancing the capture of toxic carbon dioxide, the release of breathable oxygen and production of useful biomass (Fahrion et al., 2021). In addition, their capability of using both VL and FRL for oxygenic photosynthesis, could allow the switching of photobioreactors from a high-energy demanding VL-system to a low-energy demanding FR-system, thus guaranteeing energy preservation during space travel (Liistro et al., 2024).

## Conclusion

In conclusion, the novelty of the present work is showing that in *Chroococcidiopsis* sp. CCME 010, all the far-red genes of the unusually reduced FaRLiP cluster, are transcriptionally regulated by FRL and that two closely related desert strains heterologously expressing the *chl*F^010^ gene could grow in FRL. Moreover, the fact that CCMEE 010 did not undergo an extensive PSI remodeling during FRL acclimation could explain the FRL-growth acquired by CCMEE 029 and CCMEE 064. Failure to obtain CCMEE 057 transformants in FRL deserves further investigation and may be overcome after identifying a stronger promoter and/or expression of a different *chl*F gene. In fact, varying amounts of Chl *f* were found in *Synechococcus* sp. PCC 7002 expressing *chlF* genes from different FaRLiP strains (Shen et al., 2019).

Future challenges depend on achieving a deeper understanding of the FaRLiP response in CCMEE 010 using transcriptomic and proteomic approaches. Such knowledge could not only enhance our understanding of photosynthetic diversity and adaptability but also suggest novel engineering approaches to native photosystem complexes as the specific location of the Chl *f* can be crucial in determining the photosynthetic efficiency (Mascoli et al., 2020). However, it is expected that the lack of most the FR-PSI genes in CCMEE 010 may overcome the limitations to functional integration of the Chl*f* synthase in photosystems of closely related *Chroococcidiopsis* strains.

## Data Availability

The original contributions presented in the study are included in the article/Supplementary material, further inquiries can be directed to the corresponding author.
