# Supplementary material for: Far-red light photoacclimation in a desert Chroococcidiopsis strain with a reduced FaRLiP gene cluster and expression of its chlorophyll f synthase in space-resistant isolates
Source: Front Microbiol. 2024 Sep 12;15:1450575. doi: 10.3389/fmicb.2024.1450575 (PMC11424453; doi:10.3389/fmicb.2024.1450575)
Supplement: Supplementary file 1 [file Table_1.DOCX]

| *Target gene* | *PCR primers* | *Sequence (5’-3’)* | *PCR*  *product size (bp)* |
| --- | --- | --- | --- |
|  |  |  |  |
| 16S | chr16S-F | 83 pb | TACTACAATGCTACGGACAA |
|  | chr16S-R |  | CCTGCAATCTGAACTGAG |
| *rfpA* | rfpa-F | 78 pb | TAGGTAGCACTGTCTGGTT |
|  | rfpa-R |  | AAGGCGAAGGAGGCAATA |
| *rfpB* | rfpb-F | 97 pb | TACAGCAGGAACTACAAC |
|  | rfpb-R |  | TCTGGATTATCATCAACTACT |
| *rfpC* | rfpc-F | 99 pb | AGCAGCGTCGGTTTACTGAG |
|  | rfpc-R |  | AGCGACACAAAGCCTGTTCT |
| *psaF2* | psaF02521-F | 98 pb | ATTTAGCTTTCGACCGCCCA |
|  | psaF02521-R |  | GGAACGACCACTGTAGCCAA |
| *psaJ2* | psaJ02522-F | 77 pb | TTATTGCTGTGGTTCTGATT |
|  | psaJ02522-R |  | TGGCAAAGGTTTGAAGTAG |
| *psbA4/Chlf* | chrChlF-F | 82 pb | ATCATTAACCGCGCCGATCT |
|  | chrChlF-R |  | CGCTAGTTGCTAAACCCCCA |
| *psbA3* | psbA02513-F | 94 pb | GAATCCGAGTCACAGAAC |
|  | psbA02513-R |  | AAATCAGACGACCAAAGTAG |
| *psbB2* | psbB02523-F | 85 pb | AAGATCCATTGACTGTGAA |
|  | psbB02523-R |  | TCAGAGTTGAGAGTAGCA |
| *psbD3* | psbD02519-F | 76 pb | TTGTGGATGAGTGCTGTA |
|  | psbD02519-R |  | GCAGTTCTTGGCTGATAA |
| *psbC2* | psbC02520-F | 83 pb | GGTGTAGCAACTGAACTC |
|  | psbC02520-R |  | AGGAAAGCAAAGATAAAGTGA |
| *apcB2* | apcB02515-F | 87 pb | AACATCATAACCAAGACAGTAG |
|  | apcB02515-R |  | ATAACGACGGCAAGTGTA |
| *apcD2* | apcA02516-F | 88 pb | TAGTAATAGCGGAAACAGAAC |
|  | apcA02516-R |  | CACGGCAATGGAATAAGT |
| *apcD5* | apcD_1_02514-F | 103 pb | CTATCCTGCTCCCAAAGA |
|  | apcD_1_02514-R |  | AATCCGTTGTTCGTTCTC |
| *apcD3* | apcD3_F_02518 | 92 pb | ATGAGGAGGTTCGCTATC |
|  | apcD3_R_02518 |  | AATTGTTGCTAGACGGATTC |
| *apcE2* | chr02517-F | 82 pb | GGGGCTTGCGTGGAGTTATT |
|  | chr02517-R |  | CTGAGGGACTTTCGTCGCAT |
| *apcE1* | chr04639-F | 85 pb | TCGCTGGCGATCCTAACATT |
|  | chr04639-R |  | ATTGTTGCCTCACCGGAACA |
